# The legal needs of people receiving palliative care in Uganda: A multi-method assessment to advance universal health coverage

**DOI:** 10.1177/26323524251347652

**Published:** 2025-06-26

**Authors:** Sofia Weiss Goitiandia, Eve Namisango, Emmanuel B. K. Luyirika, Faith N. Mwangi-Powell, Lynn Atuyambe, Elizeus Rutebemberwa, Paul Muhimbura, Henry Ddungu, Richard A. Powell, Fatia Kiyange, William E. Rosa

**Affiliations:** 1 Division of Hospital Medicine, School of Medicine, University of California, San Francisco, CA, USA; 2 African Palliative Care Association, Kampala, Uganda; 3 College of Health Sciences, Makerere University School of Public Health, Kampala, Uganda; 4 Uganda Cancer Institute, Kampala, Uganda; 5 Department of Primary Care and Public Health, Faculty of Medicine, School of Public Health, Imperial College London, UK; 6 Center for Health, Human Rights and Development, Kampala, Uganda; 7 Department of Psychiatry and Behavioral Sciences, Memorial Sloan Kettering Cancer Center, New York, NY, USA

**Keywords:** palliative care, serious illness, end of life, Uganda, human rights, will, Universal Health Coverage, global health, health policy

## Abstract

**Background:**

Palliative care (PC), a holistic approach to care for persons living with serious illness or injury, is a crucial component of Universal Health Coverage (UHC) and Sustainable Development Goal 3. While Uganda has made commendable progress in improving PC access, the legal aspects of PC provision remain underexplored.

**Objectives:**

Considering knowledge gaps regarding the legal aspects of PC in Uganda, this study sought to assess the legal needs and challenges faced by persons receiving PC in the country.

**Design:**

Cross-sectional design utilizing both quantitative and qualitative methods.

**Methods:**

The quantitative arm surveyed 384 individuals receiving PC across three study sites, comprising public and not-for-profit private healthcare institutions. Quantitative data were analyzed descriptively. The qualitative arm involved 25 key informant interviews conducted with healthcare providers, legal and human rights experts, and medicines supply chain professionals, along with four focus group discussions involving 40 individuals receiving PC at two study sites. Qualitative analysis was used to analyze the qualitative data.

**Results:**

Both quantitative and qualitative findings revealed significant legal challenges and practical obstacles faced by persons receiving PC in Uganda. Participants reported a lack of access to high-quality PC services, including legal assistance. Legal challenges included limited awareness of patients’ legal rights, the need for increased legal support in areas such as succession planning and will-making, and legal barriers associated with ensuring an adequate supply of opioids for pain management.

**Conclusion:**

Based on findings of unmet legal needs among individuals receiving PC in Uganda, this study provides recommendations to address these needs, strategically and pragmatically maximizing patients’ quality of life and well-being and advancing PC provision as part of UHC.

**Trial registration:**

Not applicable.

## Introduction

Palliative care (PC) is an interdisciplinary and holistic approach to care that can be delivered throughout the life course for persons experiencing serious illness and injury.^1^ PC addresses the physical, psychological, social, and spiritual needs of patients and caregivers and is fully compatible with curative treatment options at any point throughout the disease trajectory, starting at the time of diagnosis.^2^ A crucial component of ensuring person- and family-centered PC is addressing the legal and ethical dilemmas that arise in the context of serious illness to mitigate overall distress and symptom burden and optimize the quality of life while ensuring the delivery of serious illness and end-of-life care is aligned with patient and family values and preferences.^3–7^ As an essential component of Universal Health Coverage (UHC) per the World Health Organization and Sustainable Development Goal (SDG) 3,^8,9^ PC access is fundamental to the right to life, the highest possible standards of physical and mental health, and aligns with the ethics of all human rights (e.g., universality, dignity) and ensuring freedom from cruel and inhumane treatment.^10,11^

More than 70 million people worldwide experience serious health-related suffering (SHS) annually that is amenable to PC.^12^ Nearly half of the global population with SHS is near the end of life, and approximately 75% of adults in need of PC reside in low- and middle-income countries (LMICs).^13^ Despite high levels of need in LMICs, roughly two-thirds of countries have no or extremely limited access to PC services, and 83% have low to no access to medically indicated opioid medications (e.g., morphine) for pain relief.^13,14^ The global burden of SHS is estimated to nearly double by 2060 and will increase most rapidly in the world’s poorest countries.^15^

Uganda is a sub-Saharan low-income country located in East Africa. It has a national population of approximately 44.4 million, with an average life expectancy of 66.7 years as of 2019 (compared to the global average of 73.3 years).^16^ Approximately 74% of the population lives in rural areas,^17^ and about 63% are employed in agriculture.^18^ Over 40% live on less than USD 2.15 daily,^19^ and only 54% of children complete primary education.^20^ The top four causes of death in Uganda are communicable diseases—specifically, neonatal conditions, HIV/AIDS, lower respiratory infections, and malaria.^21^ These are followed by road injury, diarrheal diseases, stroke, ischemic heart disease, tuberculosis, and congenital anomalies. In terms of overall sustainable development, the country is on track to meet only about 25% of SDG targets by 2030.^22^

Uganda is among 21 countries where PC is in preliminary integration into mainstream health service provision.^13,23^ Countries in this category are characterized by: (1) a critical mass of PC activism in several locations; (2) a variety of PC provider and service types; (3) health professional and local community awareness of PC; (4) an implemented and regularly evaluated PC strategy; (5) availability of morphine and other strong opioids for pain relief; (6) some level of PC impact on policy; (7) substantial PC training and educational initiatives offered by numerous organizations; and (8) existence of a national PC organization.^13^ Uganda has been an exemplar of PC development and innovation at the intersection of policy advancement, workforce capacity building, and increased service access at the national level.^24–26^ The Ugandan Ministry of Health approved the import of morphine powder for the preparation of oral morphine liquid in 1993, followed by the government legalization of opioid prescribing by nurses and clinical officers who had been specially trained in 2004.^14,27,28^ Uganda’s investment in morphine supply and delivery has substantially improved access to persons with SHS, particularly those living in rural areas.^29^

Although nationally available and accessible pain management has been a priority and measurable deliverable of the Ugandan government, less is known about the progress of other PC domains in the country, including the legal needs of persons with serious illness. Meeting patients’ legal needs is increasingly recognized as an important component of holistic PC delivery worldwide.^3,30^ Addressing these needs has been suggested to contribute to the peace of mind, well-being, and health of patients with serious illness, ultimately improving patients’ quality of life and potentially positively impacting patients’ disease progression.^3,30,31^ Furthermore, ensuring that patients’ legal, custodial, and financial wishes are documented and honored can safeguard the livelihood and well-being of their surviving family members.^30,31^ However, significant gaps remain in our understanding of patients’ legal needs, particularly in LMICs such as Uganda, highlighting the urgency of research in this area.

To fill this gap, we conducted a multi-method study that sought to (1) quantitatively survey persons receiving PC in Uganda to identify relevant legal needs and (2) qualitatively interview persons receiving PC, healthcare providers, legal professionals, and employees in Uganda’s medicines supply chain through in-depth interviews and focus groups to further explore and contextualize the survey findings. The results have multilevel practical implications for future PC policy, education, research, and clinical practice in Uganda and the surrounding region to ensure the achievement of UHC and the SDGs.^8,9^ In particular, this study highlights the need to continue improving PC access, ensure adequate legal support to bolster the well-being and quality of life of patients receiving PC, enhance patient education regarding their legal rights and planning, and legally facilitate improved pain management, particularly through appropriate opioid access.

## Materials and methods

### Study design

This study employed a multi-method, cross-sectional design to assess the legal needs of people receiving PC in Uganda. The quantitative arm of this study consisted of a survey administered to people receiving PC. The qualitative arm, leveraged to contextualize and expand upon survey results, employed key informant (KI) interviews with healthcare providers, legal and human rights experts, and employees in the medicines supply chain, and focus group discussions (FGDs) with persons receiving PC. We report our study using the Standards for Reporting Qualitative Research guideline (Supplemental Material: S5 Appendix).

### Study setting

This study was based at three Ugandan healthcare institutions: the Uganda Cancer Institute (within Mulago Hospital), Mildmay Uganda, and Kitovu Mobile. The Uganda Cancer Institute is a publicly funded tertiary medical facility in the Ugandan capital, Kampala. It specializes in cancer care and is run in partnership with the Ugandan Ministry of Health.^32^ Mildmay Uganda, located in a peri-urban setting in the Wakiso district, is a national nongovernmental organization focused on delivering comprehensive healthcare services, with a particular focus on the needs of people living with HIV and AIDS, while also extending its reach to other populations and interventions, such as health systems strengthening.^33^ Kitovu Mobile is a not-for-profit, faith-based organization serving the Greater Masaka region, where it offers community-based HIV and AIDS preventive care and treatment, child and maternal health services, and hospice and PC.^34^ The study sites were purposively selected to maximize participant representation: the institutions represent the public and private healthcare sectors, rural and urban providers, and facilities that offer PC and general care, including services for persons living with HIV and AIDS, as well as persons with PC needs for other medical conditions.

### Study population

The present study recruited participants between January 3, 2010, and April 2, 2010. They included surveyed individuals receiving PC (*N* = 384), staff KIs (*N* = 25), and persons receiving PC who participated in the FGDs (*N* = 40 across four FGDs). For the survey component, Cochran’s formula was used to determine a sample size of 384 participants with a 95% confidence level and a 5% margin of error. Participants in the survey were individuals living with serious illness receiving PC at one of the three study sites (*N* = 128 at each site).

KIs included healthcare providers to persons receiving PC, employees of the Ugandan Ministry of Health, employees working in Uganda’s medicines supply chain (i.e., for the Ugandan National Medical Stores^35^ and the Ugandan National Drug Authority^36^), and legal officers and human rights legal practitioners working in civil society organizations.

FGD participants were persons receiving PC at Mildmay Uganda (*N* = 20) for two of the FGDs conducted and at Kitovu Mobile (*N* = 20) for the other two FGDs (total *N* of FGDs = 4). The FGDs were organized by gender into two groups of self-identifying men and two of women.

### Inclusion and exclusion criteria

For the survey, members of the local clinical teams introduced the study team to the potential study participants in outpatient settings at all the study sites. Once introduced to the study team members, potential participants were given comprehensive information about the study. All persons receiving PC who were available on the day of data collection at the relevant site, aged 18 years or older, well enough to be surveyed, and provided written consent to participate in the study were included. Individuals who were not well enough to be surveyed were cognitively impaired and could not provide informed consent, or did not provide written consent to participate, were excluded.

KIs were initially purposively sampled based on their involvement in implementing PC services in Uganda. Possible respondents working in the fields of health, patients’ rights, and legal areas as they relate to serious illness and PC delivery in Uganda were identified by the African Palliative Care Association at national, subnational, and service delivery levels. Thereafter, participants were contacted, and interested participants were introduced to the team in charge of recruitment and data collection, who scheduled interview appointments with them. Snowball sampling was then used to recruit further KIs, wherein interviewed KIs were asked about additional individuals in their networks who could be beneficial to contact for further information on legal issues affecting PC provision in Uganda. The research team subsequently contacted these individuals to request an interview.

FGD participants were purposively selected from the populations of people receiving PC at two study sites, Mildmay Uganda and Kitovu Mobile. Participants were selected to capture equal numbers of self-identified men and women over 18 living with serious illness and who could speak about the legal aspects of their illness and care. Recruited participants provided written consent to participate.

### Ethics and approvals

Ethical approval was obtained from the Uganda National Council of Science and Technology (#SS-2261). Subsequently, formal permissions to conduct the study were sought from the participating institutions’ senior management teams. Written informed consent was obtained from all participants, with consent forms translated into two local dialects of Luganda and Runyankore-Rukiga at a primary seven (12–13 years old) reading level, as recommended for research in community settings. A pretest was conducted to identify and simplify any complex language. Trained PC practitioners conducted the informed consent process with patients in facility counseling rooms. Participants were informed that participation was entirely voluntary. Additionally, a distress protocol was established to support those who showed distress during the interview and did not wish to continue. All information collected during the study has been treated as confidential, and all study data have been anonymized.

### Data collection

Data were collected from January 2010 to February 2010. The survey instrument, KI interview guides, and FGD discussion guides were collaboratively developed by the research team to elicit information relevant to the legal needs of persons receiving PC in Uganda. All tools were pretested at a fourth institution, Hospice Africa Uganda, in Kampala.^37–39^

The survey instrument included questions on participants’ sociodemographic characteristics, access to PC, and ethical and legal issues pertaining to their illness and healthcare. The survey comprised 20 closed and open-ended questions (Supplemental Material: S1 Appendix). Research assistants trained in the study’s data collection methods administered the surveys through interviews with individuals receiving PC and a paper survey instrument. The surveys were developed in English and translated by the research team into Luganda, a widely spoken indigenous language in Uganda, for delivery. The interviews took place either at the health facilities associated with the study sites or in community locations where one of the organizations provided services. The data were checked for consistency on the day of collection.

Four separate semistructured interview guides were developed for the KI interviews, aimed at healthcare providers, professionals with expertise in Uganda’s medicines supply chain, and legal and human rights experts (Supplemental Material: S2–S4 Appendices). The guiding questions addressed the availability of, and access to, PC, legal considerations within PC—including laws, conventions, and patients’ rights—the regulation of opioids, and other legal and ethical issues pertinent to PC in the Ugandan context.^3,26^ All KI interviews were conducted by the study’s principal investigators at the interviewee’s place of work. All KI interviews were conducted in English and audio recorded.

The semistructured FGD discussion guide addressed the rights of people receiving PC, their access to PC, and social and legal issues relevant to their experiences of serious illness and PC. Trained research assistants conducted the FGDs, moderating and recording the sessions, and taking notes in Luganda. The FGDs were convened at two institutions where the survey participants were recruited, with an average duration of 45 min per discussion. These sessions were also audio recorded with participants’ informed consent. Throughout the data collection phase, debriefing meetings were held at the end of each day to evaluate data quality and address emergent issues. Comprehensive summary notes were also prepared, synthesizing the content of each day’s FGDs.

### Data analysis

The survey data were cleaned and entered into EpiData software before being exported to SPSS (version 11.5) for descriptive analysis. Categorical quantitative data were summarized using proportions, and continuous data using means and standard deviations.

KI interviews were transcribed verbatim using the audio recordings and interviewer notes. The FGD audio recordings were translated verbatim into English, and the transcripts were reviewed by bilingual (Luganda–English) research team members against the audio recordings to ensure accuracy. The KI interview and FGD transcripts were read several times, with notes on salient issues, which were then discussed. This iterative process involved all members of the research team. Following an inductive qualitative content analysis approach, the transcripts were then openly coded.^40,41^ The research team iteratively organized the coded excerpts into categories and overarching themes, each supported by relevant quotations. The qualitative data analysis team met several times to ensure the themes aligned with the coded extracts, the entire dataset, and the study’s research questions. Particular attention was paid to counterfactual data to enhance rigor in theme refinement. Key quotations have been selected to illustrate the themes.

Notably, the research team comprised researchers, clinicians, and individuals with professional experience in the legal sector. Most research team members were local to Uganda and had close knowledge of PC delivery in the country, particularly among the data collection and analysis teams. Throughout the analysis, team members sought to reflexively consider how their positionalities might influence their interpretations of the qualitative data. Themes were iteratively discussed and refined among team members, drawing upon members’ diverse expertise to mitigate bias and enhance rigor.

## Results

### Sociodemographic characteristics of the participants

Three hundred and eighty-four persons receiving PC participated in the survey, with an equal representation of 128 participants from each of the three institutions sampled. Additionally, 25 KI interviews were conducted. The participants comprised eight nurses, five doctors, five legal officers, four medicine supply chain management professionals, two pharmacists, and one sociologist. The FGD participants included 20 self-identified men and 20 self-identified women who disclosed living with serious illness.

Nearly a third (58.6%) of those receiving PC surveyed self-identified as women. The majority (68.5%) of survey participants were between 25 and 45 years old. Most of the participants’ highest level of education was upper primary (28.6%) or lower secondary (29.7%) education. Most participants (80.2%) had a primary diagnosis of either HIV/AIDS (58.6%) or cancer (21.6%). Only two individuals (0.5% of the sample) were not willing to disclose their primary diagnosis. The range of illness duration was 1–21 years. 30.5% of participants had been ill for 2 years or less, and approximately three-quarters (74.8%) reported having been ill for 5 years or less. Further, most persons receiving PC (88.6%) had spent 5 years or less on a PC program. Across all primary diagnoses, the median survival was 2 years, and the mean was 4.41 years.

Table 1 summarizes the survey participants’ sociodemographic characteristics.

**Table 1.** Sociodemographic characteristics of the survey participants.

Characteristic	Variable	Frequency (*n* = 384)	Percentage
Gender	Men	159	41.4
	Women	225	58.6
Age group (years)	18–24	24	6.3
	25–35	120	31.3
	36–45	143	37.2
	>45	97	25.3
Marital status	Single	85	22.1
	Married	104	27.1
	Divorced or separated	56	14.6
	Widowed	102	26.6
	Cohabiting	37	9.6
Education level	None	40	10.4
	Lower primary (years 1–4)	55	14.3
	Upper primary (5–7)	110	28.6
	Lower secondary (1–4)	114	29.7
	Upper secondary (5–6)	15	3.9
	Tertiary	50	13.0
Reported illness	Cancer	83	21.6
	HIV/AIDS	225	58.6
	Other conditions	74	19.3
	Not willing to disclose	2	0.5
Illness duration (years)	1	46	12.0
	2	71	18.5
	3	59	15.4
	4	50	13.0
	5	61	15.9
	6	26	6.8
	7	23	6.0
	>7	48	12.5
Time spent on a PC program (years)	1	87	22.7
	2	101	26.3
	3	73	19.0
	4	48	12.5
	5	31	8.1
	6	17	4.4
	7	7	1.8
	>7	20	5.2

PC: palliative care.

Additional findings from the quantitative and qualitative arms of this study are categorized by access to PC—a precursor to receiving legal support in many cases—persons receiving PC’s awareness of their human and legal rights, PC recipients’ need for legal support in inheritance disputes and in making a will, and legal and practical challenges to navigating opioid access in the Ugandan context.

### Access to PC

People with PC needs in Uganda face several challenges in accessing care, the downstream effects of which can include limiting their access to support to help fulfill their legal needs. Study participants specified that barriers include a lack of knowledge of existing PC—including legal support—services, among persons with PC needs, individuals living long distances from services, and late referral to PC services by healthcare professionals. KIs interviewed suggested that, as PC is not commonly offered in primary healthcare facilities, some people do not know it is available in other facilities. When individuals are aware of the facilities offering PC services, participants in the survey, KIs, and FGDs indicated that these individuals are often unable to access those services because of the long distances from their homes to facilities where services are located. Persons receiving PC included in the FGDs highlighted that people who could benefit from PC services are often not strong enough to walk to facilities. Further, survey responses showed that transport cost also represents a barrier to accessing PC services. Almost half (47.7%) of the participants surveyed reported incurring transport costs of more than USD 2.50—more than 1 week of treatment for 68.8% of participants—to get from their homes to their treatment facility (Table 2). In line with this finding, most KIs indicated that poverty was a key impediment to Ugandans accessing PC, including legal support services. The specific PC services sought by the survey participants in this study are outlined in Table 3.

**Table 2.** Cost of one-way transport to a PC health facility compared to treatment costs for 1 week of PC treatment.

Cost (USD)	Cost of transport		Cost of 1 week of PC treatment	
	Number of survey participants (*N* = 384)	Percentage of survey participants (%)	Number of survey participants (*N* = 384)	Percentage of survey participants (%)
⩽2.5	183	47.7	264	68.8
2.5 ⩾ 5	119	31.0	6	1.6
>5	82	21.4	114	29.7

PC: palliative care.

**Table 3.** PC services sought by survey participants.

Service	Number (%)*
Pain control	257 (66.9)
Spiritual support	85 (22.1)
Legal assistance	70 (18.2)
Nutritional counseling	67 (17.4)
Counseling on medication adherence	34 (8.9)
Prevention education on infection control	36 (9.4)

PC: palliative care.

* Participants were permitted to provide more than one answer.

Over 18% of survey respondents indicated seeking legal assistance as part of their PC. Notably, 66.9% of survey participants reported that they received treatment for pain. Of those who did not receive pain treatment (33.1%), 79.5% reported not experiencing pain—leaving 20.5% of participants whose pain may have gone uncontrolled.

Though services were reported to make efforts toward a holistic approach to care, both FGD and KI participants disclosed a lack of standardized quality of PC services offered by healthcare facilities. For instance, the availability of legal assistance was reported to be contingent on facilities’ professional networks, as legal services are sourced externally. Furthermore, even within a single facility, the consistency of care was suggested to vary. For example, participants mentioned that access to PC medicines, especially opioids, can hinge on contributions from third-party donors and the presence of healthcare providers with the necessary authorization to prescribe and administer these medications to persons with specific conditions. Consequently, if an individual presents to a facility on a day when the relevant healthcare worker(s) are unavailable, they may be unable to access the medications they need.

### Awareness of human and legal rights among persons receiving PC

FGD participants were asked about their awareness of their human and legal rights and the issues they faced concerning them. Similarly, KIs were probed about their perceptions of the rights of persons receiving PC and whether these were reflected in Ugandan laws pertaining to PC and in individuals’ medical treatment. FGDs uncovered a high level of awareness among participants regarding their human rights, such as their right to property, employment, healthcare access, and equality and nondiscrimination (Quotation 1, Table 4).^42–44^

**Table 4.** Quotation table from KI interviews and FGDs with persons receiving PC.

Quotation number	Relevant finding	Example quotation
1	Persons receiving PC’s awareness of their human and legal rights	“*As a mother, I have a right to inherit property that my husband left behind upon his death. Though I have the virus, I have a right to work. Nobody should discriminate against me on the grounds that I have the virus because I am a human being like any other person*.” (FGD, Woman)
2	Persons receiving PC’s awareness of their human and legal rights	“*They are supposed to consent but most of them [patients] don’t know their rights. Out of 10 patients, about two know their rights*.” (KI, Medical Officer)
3	Persons receiving PC’s need for legal support: inheritance disputes	“*From my point of view, the law is very weak to protect the children or the widow upon the death of the head of household. Personally, I was given land in the village but when my husband died, my mother-in-law took it*.” (FGD, Woman)
4	Persons receiving PC’s need for legal support: inheritance disputes	“*Women also suffer a lot when it comes to violating their rights especially after the death of their husbands. The relatives come in and take all the property that was left behind. The law can’t protect these people who are left behind so that their rights are not violated*.” (FGD, Man)
5	Persons receiving PC’s need for legal support: inheritance disputes	“*Right now, she is in the village selling all the property that belongs to the children. I don’t have anywhere to run to for redress because I am also scared. There is no one to help me at all, so my request is that we be helped in such situations. I can’t even report her anywhere because she claims she was related to my husband and is the rightful owner of that property*.” (FGD, Woman)
6	Persons receiving PC’s need for legal support: making a will	“*Certainly, testate succession [with a valid legal will] is better than intestate succession [without a valid legal will]—it minimizes land wrangles, etc. You leave more confusion if you die intestate, and the confusion is resolved against the weak, which in our cultural settings are mostly the women, children, and, I should say, the terminally ill, because they are physically weak*.” (KI, Legal Officer)
7	Persons Receiving PC’s need for legal support: making a will	“*I think it is historical that we regard that [succession planning] as being private. If you start asking about somebody’s property, you may be misunderstood, and people may think you have an interest. So, most of my work has been around caring for patients unless the family brings it up, but we try not to. We may advise during the period of care, but we do not go into details of who will take what, where the children will be, etc. I think this [succession planning] is a good thing, which we can include in our program, but it is quite sensitive*.” (KI, Medical Officer)
8	Persons receiving PC’s need for legal support: making a will	“*We don’t talk about it [making a legal will] because whenever people think about a will, they think about death. So, we don’t talk about it. Yes, it is very important to these patients [. . .] but we can’t bring it up unless they have brought it up themselves*.” (KI, Nursing Officer)
9	Persons receiving PC’s need for legal support: making a will	“*I think the will helps a lot. We are four women, so if the man can divide the land and leave every woman with a piece of land to stay on with the children, this helps a lot in avoiding disputes*.” (FGD, Woman)
10	Persons receiving PC’s need for legal support: making a will	“*If a will is done, all these disputes will be avoided because it will be written that: ‘My wife should never be chased from here; my property will be given to so and so’. You find that the relatives will respect such a thing and the woman won’t be chased away*.” (FGD, Woman)
11	Legal and practical challenges to navigating opioid access	“*Due to limited funds, opioids are not a priority when it comes to procurement. In the international market, there is a limited scope. The organizations that can supply high-quality opioid medicines are very few. Distance is also a problem; those narcotic medicines are sourced from different stores in Europe and America. The imported medicines have to be registered with the National Drug Authority upon payment of a retention fee that depends on the quantity, but this escalates the costs and eventual market price*.” (KI, Medicines Procurement Officer)
12	Legal and practical challenges to navigating opioid access	“*There may be worries because we don’t know what happens at home. At times, some of these patients are given a bottle of morphine to go home with and we don’t know what takes place there*.” (KI, Medical Officer)
13	Legal and practical challenges to navigating opioid access	“*All those strong pain-relieving medicines are available and allowed to be dispensed but are restricted. As the National Drug Authority (NDA), we are the drug regulatory agents of the Government and that includes the regulation of all classified medicines. Although certain medicines are strictly controlled by the NDA, we ensure they are available but only dispensed by registered and authorized institutions and personnel*.” (KI, Medicines Inspector)

PC: palliative care.

Nonetheless, KI interviews with healthcare providers and FGDs suggested that individuals receiving PC are not always aware of their legal rights concerning medical treatments (Quotation 2, Table 4). These include their right as patients to provide informed consent for any treatment they undergo.^45^ Additionally, participants noted that, in numerous instances, such rights fail to be met; for example, participants noted that patients frequently do not receive comprehensive medical information and are not required to sign consent forms.

### Need for legal support among persons receiving PC: Inheritance disputes

Participants raised several legal issues faced by persons receiving PC and their families. One key issue concerned inheritance and associated land disputes. Even though many participants recognized that spouses and children hold a legal right to inherit property—this has been the case under the Ugandan Constitution since 1995^46,47^—they suggested families often encounter obstacles in securing their inheritance rights following the death of the person receiving PC, particularly if they were the head of household (Quotation 3, Table 4).

Participants pointed out that widowed individuals, especially women, frequently confront the possibility of their property being seized following the death of their spouse, often by their spouse’s relatives. Some participants framed this issue in light of their perception that, in practice, the law alone cannot guarantee the protection of rights—especially property rights—for the widowed spouses and children of persons receiving PC (Quotations 3 and 4, Table 4). These participants expressed their frustration with the limited options available to them to prevent such actions on the part of, for example, their spouse’s relatives (Quotations 4 and 5, Table 4).

### Need for legal support among persons receiving PC: Making a will

The legal aspects of preparing for the death of a person receiving PC were of widespread concern, including help needed in making a will (e.g., for the proper transfer of property and bereaved family support). Uganda’s Succession Act, last amended in 2022, permits anyone with mental capacity to dispose of their property legally.^48^ Wills assist individuals in ensuring their resources benefit their surviving family members following their wishes. KIs indicated that this legal aspect of PC could alleviate individuals’ psychological suffering and potential social conflicts associated with succession disputes (Quotation 6, Table 4).

However, participants indicated that persons receiving PC face several obstacles to making a valid will. Healthcare provider KIs suggested that many people are unaware of how to make a will. They added that raising the subject of wills with persons receiving PC poses difficulties, both because discussing property can lead to provider–patient misunderstandings and because of cultural taboos concerning discussing death (Quotation 7, Table 4). Thus, providers may wait for the individuals they are treating to initiate the discussion themselves (Quotation 8, Table 4).

Moreover, for persons receiving PC who do seek to make a will, legal advocates to support creating them were reported to charge high fees, rendering their services unaffordable to many—including those who may already be financially strained by their treatment and associated costs, such as travel expenses (Table 2). Only some of the facilities in this study supported persons receiving PC with making a will. Further, KI respondents related stringent legal requirements that must be followed for a will to be valid, meaning that wills frequently fail to meet them and are dismissed in court. KIs suggested that most Ugandan persons receiving PC die intestate, that is, without a valid will.

Participants recounted that intestate succession could exacerbate property disputes after a person receiving PC’s death. Though succession law in Uganda offers guidance for the distribution of property when a person dies intestate, with the widowed spouse and children the first to receive property,^48^ KIs suggested this is regularly disregarded. KIs framed this not solely as an issue with the law itself but also with customary practices that fail to recognize the rights of spouses, especially women, and children, to control the property of the deceased, which hinders the application of the law.

KI and FGD participants emphasized the potential for creating a will to mitigate these challenges, but they held mixed views about the effectiveness of wills in practice. KIs, such as legal officers, outlined instances of nonadherence to wills as rooted in cultural traditions, including the belief among some people that the relatives of the deceased can override a will, as well as a lack of legal mechanisms to enforce wills. Still, several FGD participants shared that it was easier to assume land ownership if the deceased had made a will (Quotations 9 and 10, Table 4).

Of the persons receiving PC surveyed, although 89.3% (343/384) were familiar with the concept of wills, only 34.4% (132/382) had completed any succession planning, with a marginally higher percentage of self-identifying men (38.4%, 61/159) receiving advice on succession planning than self-identifying women (31.6%, 71/225).

### Legal and practical challenges of navigating opioid access

Participants—particularly KIs—pointed out the enduring challenges, including legal ones, faced in supplying persons receiving PC with PC medicines, especially opioids. Opioid use in Uganda adheres to a strict regulatory framework guided by both national policies and international directives, such as the Single Convention on Narcotic Drugs.^26,49–51^ This international treaty obliges countries to submit estimates of their annual use to the International Narcotics Control Board, necessitating close monitoring of procurement.^26,49^ Opioids are delivered to public healthcare institutions by the National Medical Stores and to private institutions by the Joint Medical Stores, both of which track distribution.^26,52,53^ KIs interviewed indicated that Ugandan healthcare facilities must be accredited to supply opioids to patients—accreditation is awarded by the Palliative Care Association of Uganda and the Ministry of Health^26,54^—and that receipt of these medications by facilities requires the signature of a health facility director or store managers to ensure against their diversion.

KIs mentioned the challenges that persist in opioid procurement, indicating that these are primarily financial at the national level (Quotation 11, Table 4). KIs also highlighted the challenge of inadequate coordination in the procurement and distribution of opioids among national suppliers, which can create obstacles for healthcare facilities in obtaining sufficient supplies. Furthermore, under Ugandan law, only doctors and nurses with specialized training are permitted to prescribe and dispense opioids,^26,28,55^ with KIs suggesting there is a shortage of prescribers to meet the need for PC opioids.

Nonetheless, several KIs within the medicine supply chain emphasized the necessity of restrictions to limit the use of opioids. They justified these restrictions based on the perceived dangers of opioids in their potential for dependence (Quotation 12, Table 4). KIs underscored their belief in the importance of reserving opioid access for authorized institutions and individuals with a demonstrated need (Quotation 13, Table 4).

## Discussion

A lack of evidence regarding the legal needs of Ugandan persons living with serious illness prevents the advancement of PC provision and integration, hinders the efficacy and sustainability of available PC services, and slows Uganda’s progress toward UHC and SDG 3. This study set out to fill this knowledge gap and rigorously evaluate the legal needs of individuals receiving PC in Uganda by eliciting the key stakeholder perspectives of persons receiving PC, providers working in the healthcare system, and legal and medical supply chain professionals. Both the quantitative and qualitative findings identified high-level legal challenges and practical obstacles faced by people receiving PC in Uganda. Results centered on limited access to PC services—including relevant legal support—the need for legal assistance in areas such as succession planning and will-making, and the enduring legal challenges associated with ensuring a sufficient supply of opioids for pain management.

Barriers to accessing PC included a limited awareness of available PC services—including legal ones—and large geographical distances to healthcare facilities. These challenges were exacerbated by the financial burden of transportation costs, consistent with studies indicating a lack of affordable transport as a significant reason for Ugandans delaying medical treatment or missing appointments.^26,56^ These hurdles in accessing PC parallel barriers observed in other healthcare services, resulting in delayed diagnoses and treatment initiation; for example, evidence from various types of cancers, including breast, cervical, and lung cancer, indicates that a majority of Ugandans present to providers with advanced stages of disease and high levels of need for services.^57–61^ This is common in Uganda and several other sub-Saharan African contexts, where health services, especially primary care, remain limited.

Additionally, despite healthcare facilities’ efforts to deliver comprehensive PC once individuals managed to reach them, the participants in this study pointed to a lack of standardized quality across different facilities, resulting in experiences of inconsistent service provision. Relevant issues raised encompassed variability in the provision of legal assistance and in the availability of medications, particularly opioids, including due in part to the limited number of healthcare providers authorized to prescribe them at different facilities.^55^

Concerning the human and legal rights of individuals receiving PC, many participants displayed high awareness of their human rights, including the right to property, employment, healthcare access, and equality and non-discrimination.^42–44^ However, knowledge of legal rights concerning medical treatments, such as the right to informed consent, was reported to be more limited.^45^ Furthermore, having a high level of knowledge did not necessarily translate into fulfilling individuals’ legal rights and needs, as external factors often hindered their realization. For example, despite recognizing the importance of succession planning and will creation among many participants, only a few had the opportunity to create a will, and some KIs questioned the efficacy of a will in ensuring its enforcement. Such challenges in guaranteeing the legal needs of individuals receiving PC, such as property transfer in alignment with their preferences, may adversely affect their psychological and social well-being, as they experience a limited ability to guarantee that their loved ones will be taken care of after their death.^3^

Many of the legal issues addressed by participants exhibited distinctly gendered dynamics that intertwine with broader struggles for gender equity in the Ugandan context (e.g., asset ownership, access to economic resources, social protection, freedom from stigma and violence).^62–65^ For example, participants addressed the legal and cultural obstacles women face, particularly regarding succession planning and property transfer. Cultural norms and customary practices were identified as factors contributing to the marginalization of women, especially widowed individuals, and children in matters of property inheritance, particularly when the deceased was considered the head of the household. Women, especially if widowed, were reported to encounter the risk of losing any property they inherited, as relatives asserted their claims to the deceased’s assets. Participants stressed women’s minimal access to legal recourse in such situations.

The challenges reported regarding women’s experiences related to property inheritance and ownership are reflected in the broader literature and contribute to keeping women in economically and socially disadvantaged positions compared to men in their communities and society at large, hindering the achievement of gender equity in Uganda.^66–69^ While recent legal reforms, such as Uganda’s amended Succession Law in 2022, aim to enhance gender equity in laws such as succession planning by granting greater legal rights to women and children,^48,70,71^ the gap between policy and practice remains a challenge.^67,72^ Further advocacy, social change, and increased access to legal support specifically for women will likely be necessary to realize fully the intended benefits of new legislation, including for women who experience a spouse’s death.^73^

The destigmatization of the end of life and PC in Uganda is also crucial for securing the legal needs of persons receiving PC. Such shifts require clinicians to be equipped with advocacy skills to communicate effectively with the public, decision-makers, and nonpalliative/generalist clinicians regarding the benefits and outcomes related to PC, policies supporting PC development and implementation, and the clarification of PC misinformation.^74–76^ Cultural and societal taboos have historically shrouded discussions surrounding death and end-of-life decisions,^77–79^ which may inform some of the difficulties reported by participants in engaging in conversations about will creation and succession planning. Reducing the stigma surrounding these conversations may help people articulate their wishes, access will creation services, and provide for their loved ones in a legally recognized manner. This, in turn, may minimize potential legal ambiguities and property disputes among family members, alleviating the psychosocial burden on individuals receiving PC and their families.

Similarly, broad legal and social factors continue to shape the provision of opioids for pain management to persons receiving PC in Uganda. While alleviating physical pain is only one facet of holistic PC provision, the availability of opioids remains an important benchmark for assessing PC services worldwide.^14,26,80^ Though this study did not focus on quantifying the availability of opioids for PC in Uganda, participants did name enduring barriers to obtaining opioids. These included procurement difficulties, partially attributable to international and national regulations governing opioids, as well as a shortage of healthcare providers authorized to prescribe opioids to persons receiving PC. Nevertheless, many respondents advocated in favor of stringent opioid restrictions due to concerns about their misuse.

These contrasting perspectives reflect ongoing debates in Uganda about how accessible opioids should be to patients in general. On the one hand, Uganda’s National Drug Policy and Authority Act obliges the National Drug Authority to ensure the availability of essential medicines to the Ugandan population, including opioids for PC, as on the World Health Organization’s Model List of Essential Medicines.^81^ Access to medically and scientifically indicated morphine is strongly supported by the World Health Organization and other leading global health bodies advocating for comprehensive PC access as an integral component of UHC.^14,26,80^ The World Health Organization’s 2023 “Left Behind in Pain” report advocates for universal access to generic morphine in healthcare^80^; this call resonates with the appeal for access to morphine in PC articulated by the Lancet Commission on Palliative Care and Pain Relief’s 2018 report.^14^ Additionally, recent data attest to inadequate pain management as a key factor correlated with a low quality of death, as perceived by the caregivers of persons receiving PC in Uganda.^82^ On the other hand, legal restrictions on opioids in Uganda,^55,83^ along with attitudes in favor of them, are informed by long-standing concerns about preventing their misuse.^50,51,84–86^ Yet, when such restrictions result in unmet needs for individuals receiving PC,^51^ they may be seen as limiting the fulfillment of Ugandans’ right to PC within the context of their right to health.^87,88^

Despite significant progress in PC provision, Uganda continues to grapple with the challenge of prioritizing PC amidst competing, unmet needs within its essential healthcare services.^26,52,89^ There is a pressing need for increased advocacy efforts to underscore PC as an integral part of healthcare and ensure its comprehensive inclusion in healthcare services.^26,84,86^ In line with previous research, this study’s survey findings indicate that most persons receiving PC in Uganda have a primary diagnosis of either HIV/AIDS or cancer.^54,90–92^ Therefore, it is also imperative to build greater awareness of the benefits of PC for individuals living with other conditions to broaden access to PC services, including legal ones. These changes will be of most benefit if they are coupled with enhanced monitoring and evaluation of PC services. Though international treaties obligate member states to harmonize their health-related laws and policies with international human rights standards,^42^ a lack of effective local-level monitoring mechanisms may hamper the enforcement of these legal commitments in Uganda.^54,93^ There is a need for improved monitoring and evaluation mechanisms for PC policies, delivery, and quality in the Ugandan context; such measures have the potential to assess and promote the quality of PC services more effectively.^54,93^

### Study strengths and limitations

This study has several strengths. To our knowledge, it is one of the few studies to shed light on the legal needs of persons receiving PC in Uganda, a previously underexplored issue in the literature. This study also leveraged both quantitative and qualitative data derived from a wide range of participants—spanning individuals receiving PC, healthcare providers, legal officials, and professionals working in Uganda’s medicines supply chain—to gain a deeper understanding of the legal needs of people receiving PC in Uganda than is afforded through one research method or from one stakeholder’s perspective alone. Further, sampling individuals receiving PC across various public, nongovernmental, and faith-based, as well as urban and rural healthcare facilities, may increase the generalizability of the findings.

There are also several limitations; the most notable is the presentation of data collected over a decade ago. Despite this important limitation, little has changed in terms of policy in Uganda since the study was conducted, suggesting that our results continue to pose myriad implications for the future of PC research and practice in the country.^13^ The provision of PC in Uganda and across many other sub-Saharan countries remains restricted.^13^ This PC and pain relief “access abyss” has been termed a “moral travesty” by international experts,^14^ and legal support for the Ugandan population living and dying with serious illness continues to be insufficient. Though our results remain relevant in the country’s PC capacity, they must be interpreted within current legal discourse and emerging data on the burden of suffering and PC availability.^13^ Moreover, the limited dissemination of research conducted in sub-Saharan countries, including Uganda—particularly research led primarily by local teams—may constrain the implementation of evidence-based healthcare policy and practice improvements.^94–96^ We emphasize the importance of disseminating the present research findings in this regard.

An additional limitation is this study’s geographical scope, which is confined to three districts within Uganda, all in its central region: the capital city, Kampala, the Wakiso district, and the Greater Masaka region. This may limit the applicability of the results to other areas of the country, especially as it is well documented that a high proportion of Ugandan PC services are concentrated in Kampala and surrounding districts, including Wakiso.^26^ Further, this study did not include private for-profit healthcare providers, despite these playing an important role in healthcare delivery in Uganda.^26,97^ Future studies could examine differences in individuals receiving PC’s experiences and legal needs across public, private not-for-profit, and private for-profit healthcare settings, as well as among those patients receiving outpatient versus inpatient PC (as this study focused on outpatient care).

Furthermore, our study’s limited geographical scope may also restrict the generalizability of its findings to other sub-Saharan countries with different PC contexts. Replicating this study beyond Uganda would help build stronger regional evidence on the legal needs of patients receiving PC across sub-Saharan Africa.

From a methodological standpoint, the translation of research instruments and FGDs between Luganda and English introduces the possibility of variations in conveying participants’ original or intended meanings, which may impact the interpretation of the results. Moreover, the recruitment strategy for persons receiving PC, which focused exclusively on recipients at the designated study sites, may overlook the legal needs of individuals requiring PC but who may not have been able to access such services. Lastly, this study focused on the legal needs of people receiving PC, with limited exploration of the corresponding needs of their families and healthcare providers. Therefore, further research focusing on families and healthcare providers is warranted to achieve a more comprehensive understanding of the legal needs of the various stakeholders involved in caring for persons receiving PC in Uganda.

### Implications and recommendations

Based on our findings, we provide several recommendations for different stakeholders (e.g., policymakers, healthcare providers, etc.) to better address the legal needs of people receiving PC in Uganda, as described in our study. These recommendations are outlined in Panel 1.

**Panel 1.** Recommendations for addressing the legal needs of people receiving PC in Uganda.

Relevant finding	Recommendation	Stakeholders and action areas
Access to PC	1: Work across entities, such as the Ministry of Health, the Palliative Care Association of Uganda, and other stakeholders to support the implementation of PC standards, for example, the African Palliative Care Association’s Standards for the Provision of Palliative Care, to ensure delivery of high-quality care and consistency of care across facilities.^98^ 2: Integrate PC services at the primary level of healthcare to reduce transportation costs incurred and increase individuals’ access to services, including legal ones—and so fulfillment of their human right to health. This goal synergizes with Uganda’s National Community Health Strategy, launched in 2022, which calls for building the capacities of lower-level health centers as well as village health teams and Community Health Workers to provide health services at household and community levels.^99^ 3: Integrate PC messaging in national health information and awareness programs of the Ministry of Health, for example, through the Education and Health Promotion Division, to compliment NGO-led awareness initiatives. 4: Increase awareness and advocacy efforts regarding the benefits of PC, including for persons with diagnoses other than HIV/AIDS and cancer, to improve access among all populations who could benefit from PC services, such as legal ones.	1: National policymaking; healthcare organization; and governmental and nonprofit partnerships. 2: National and local policymaking; healthcare organization; healthcare workforce capacity; and governmental and nonprofit partnerships. 3: National policymaking; education for providers and patients; government and nonprofit partnerships. 4: Education for providers and patients and Civil society advocacy.
Persons receiving PC’s awareness of their legal rights	5: Having identified some specific legal needs among persons receiving PC, provide additional training to healthcare providers and accessible resources to individuals receiving PC about their legal rights when undergoing medical treatments. Resources to support this objective could include 2019’s Patient Rights and Responsibilities Charter^100^ and the African Palliative Care Association’s legal and human rights resources for patients, caregivers, and healthcare workers.^101^	5: Healthcare delivery; education for providers and patients; and healthcare workforce capacity.
Legal support with will-making	6: Prioritize and increase the provision of legal support available to individuals receiving PC in healthcare facilities, especially support related to identified needs concerning will-making and succession planning activities. 7: Organize learning exchanges with PC programs that exemplify best practices in providing legal support, such as will-making assistance, to facilitate the expansion of such support to other healthcare facilities delivering PC.	6: Access to legal counsel; healthcare delivery; and access to legal counsel. 7: Healthcare delivery; access to legal counsel; education for providers and patients; and healthcare workforce capacity.
Legal support with property disputes	8: Advocate for free legal aid services for individuals and families receiving PC unable to afford the services of lawyers. This could involve establishing as well as sustaining existing legal support networks, including organizations providing legal assistance around specific areas such as land disputes, HIV/AIDS, and more. 9: Advocate for gender-responsive PC services that are cognizant of the most structurally marginalized groups in society, including women, and put in place interventions to address legal needs that are unique to such groups (e.g., pragmatic challenges in effective succession, as described in this study). 10: Expand availability and awareness of the legal resources available to individuals, particularly widowed women and children, facing property disputes after the death of a person receiving PC.	8: Access to legal counsel; Civil society advocacy; and education for providers and patients. 9: Healthcare delivery; healthcare workforce capacity; Civil society advocacy; and education for patients and providers. 10: Access to legal counsel and Civil society advocacy.
Opioid management	11: Continue and expand sensitization efforts—at the healthcare provider, policymaker, and public levels—regarding the appropriate use of opioids for pain in people receiving PC using the best available evidence and internationally recognized standards. 12: Train more prescribers to prescribe opioids and support the equitable distribution of their services across the country. 13: Advocate for policies and measures—including legal ones—to streamline the opioid procurement process and secure adequate distribution of these medicines to all Ugandan centers providing PC, in line with calls for universal access to generic morphine issued by the World Health Organization and other global health bodies.	11: Policymaking; healthcare organization; Civil society advocacy; and education for providers and patients. 12: Healthcare workforce capacity and healthcare organization. 13: Policymaking; healthcare organization; and governmental and nonprofit partnerships.
*To support the above areas*: legal oversight of PC	14: Explicitly include PC in national and local legal and policy frameworks related to achieving UHC, SDG3, and the fulfillment of Ugandan citizens’ right to health. 15: Enhance monitoring and evaluation mechanisms for PC to ensure its delivery aligns with international human rights and legal standards.	14: Policymaking and governmental and nonprofit partnerships. 15: Policymaking; healthcare organization; and governmental and nonprofit partnerships.

PC: palliative care; SDG: Sustainable Development Goal; UHC: Universal Health Coverage.

## Conclusion

Uganda has made commendable progress toward PC access for serious illness, yet legal needs remain understudied. Our multi-method study identified legal and practical challenges facing Ugandans receiving PC, including limited awareness of legal rights, inadequate support for will-making and property inheritance, and legal restrictions on essential PC medicines, namely, opioids. Alongside legal needs, our findings underscore the need for improved, affordable, and consistent PC. We provide recommendations for healthcare, legal, and policy stakeholders to address outlined needs to enhance patients’ well-being and advance PC provision. Notably, our findings must be interpreted in the context of their age, emerging legal discourse in Uganda, and more recent data. We stress ongoing efforts to raise PC awareness, ensure legal protections, and expand legal assistance for patients and families. Addressing these gaps is crucial for strengthening PC services, achieving UHC and SDG 3, and upholding the legal rights of those facing serious illness.

## Supplemental Material

sj-docx-1-pcr-10.1177_26323524251347652 – Supplemental material for The legal needs of people receiving palliative care in Uganda: A multi-method assessment to advance universal health coverage Supplemental material, sj-docx-1-pcr-10.1177_26323524251347652 for The legal needs of people receiving palliative care in Uganda: A multi-method assessment to advance universal health coverage by Sofia Weiss Goitiandia, Eve Namisango, Emmanuel B. K. Luyirika, Faith N. Mwangi-Powell, Lynn Atuyambe, Elizeus Rutebemberwa, Paul Muhimbura, Henry Ddungu, Richard A. Powell, Fatia Kiyange and William E. Rosa in Palliative Care and Social Practice

sj-docx-2-pcr-10.1177_26323524251347652 – Supplemental material for The legal needs of people receiving palliative care in Uganda: A multi-method assessment to advance universal health coverage Supplemental material, sj-docx-2-pcr-10.1177_26323524251347652 for The legal needs of people receiving palliative care in Uganda: A multi-method assessment to advance universal health coverage by Sofia Weiss Goitiandia, Eve Namisango, Emmanuel B. K. Luyirika, Faith N. Mwangi-Powell, Lynn Atuyambe, Elizeus Rutebemberwa, Paul Muhimbura, Henry Ddungu, Richard A. Powell, Fatia Kiyange and William E. Rosa in Palliative Care and Social Practice

sj-docx-3-pcr-10.1177_26323524251347652 – Supplemental material for The legal needs of people receiving palliative care in Uganda: A multi-method assessment to advance universal health coverage Supplemental material, sj-docx-3-pcr-10.1177_26323524251347652 for The legal needs of people receiving palliative care in Uganda: A multi-method assessment to advance universal health coverage by Sofia Weiss Goitiandia, Eve Namisango, Emmanuel B. K. Luyirika, Faith N. Mwangi-Powell, Lynn Atuyambe, Elizeus Rutebemberwa, Paul Muhimbura, Henry Ddungu, Richard A. Powell, Fatia Kiyange and William E. Rosa in Palliative Care and Social Practice

sj-docx-4-pcr-10.1177_26323524251347652 – Supplemental material for The legal needs of people receiving palliative care in Uganda: A multi-method assessment to advance universal health coverage Supplemental material, sj-docx-4-pcr-10.1177_26323524251347652 for The legal needs of people receiving palliative care in Uganda: A multi-method assessment to advance universal health coverage by Sofia Weiss Goitiandia, Eve Namisango, Emmanuel B. K. Luyirika, Faith N. Mwangi-Powell, Lynn Atuyambe, Elizeus Rutebemberwa, Paul Muhimbura, Henry Ddungu, Richard A. Powell, Fatia Kiyange and William E. Rosa in Palliative Care and Social Practice

sj-docx-5-pcr-10.1177_26323524251347652 – Supplemental material for The legal needs of people receiving palliative care in Uganda: A multi-method assessment to advance universal health coverage Supplemental material, sj-docx-5-pcr-10.1177_26323524251347652 for The legal needs of people receiving palliative care in Uganda: A multi-method assessment to advance universal health coverage by Sofia Weiss Goitiandia, Eve Namisango, Emmanuel B. K. Luyirika, Faith N. Mwangi-Powell, Lynn Atuyambe, Elizeus Rutebemberwa, Paul Muhimbura, Henry Ddungu, Richard A. Powell, Fatia Kiyange and William E. Rosa in Palliative Care and Social Practice
