# Supplementary material for: The legal needs of people receiving palliative care in Uganda: A multi-method assessment to advance universal health coverage
Source: Palliat Care Soc Pract. 2025 Jun 26;19:26323524251347652. doi: 10.1177/26323524251347652 (PMC12202919; doi:10.1177/26323524251347652)
Supplement: sj-docx-1-pcr-10.1177_26323524251347652 – Supplemental material for The legal needs of people receiving palliative care in Uganda: A multi-method assessment to advance universal health coverage [file sj-docx-1-pcr-10.1177_26323524251347652.docx]

**S1 Appendix:**

**Survey Tool for Assessing the Legal Needs of Persons Receiving Palliative Care**

**Survey questionnaire for patients:**

***The interview is done in a place where the client feels comfortable. First an informed consent is sought from the in-charge of the organisation but also the client or the attendant if the client is a child. The sheet containing information about the study and seeking the client’s consent is given to the client.***

Respondent no: ________________ Organisation: ______________________

Date: ___________________ Interviewer’s name: _______________________

**Section A: Background characteristics of the respondents:**

1. Sex: 1) Male 2) Female

2. How old are you: ______________

3. Which is your current home district? _____________

4. Marital status: 1) Single 2) Married

3) Divorced / Separated 4) Widowed

5) Cohabiting

5. What is your highest level of education?

1) None

2) Primary 1 – 4

3) Primary 5 – 7

4) Secondary 1 – 4

5) Secondary 5 – 6

6) Tertiary

6. Have you ever been told by a doctor or any health worker what illness you have?

1) Yes

2) No (Go to No 8)

7. Would you be willing to tell me what your illness is?

1) Cancer of any kind

2) HIV/AIDS

3) Any other condition, specify. _______________

4) Not willing to disclose

8. How long have you had this illness? ________________

9. How long have you been part of this programme under this facility? __________ Probe *the relationship between the length of illness and time in programme – referral, symptoms, etc.*

**Section B: Access to health care**

10. How much does it cost you to come from your home to this place to get treatment? ___________________ (transport, lunch, etc.)

11. How often do you come to the facility?

12. How much do you spend on medicines in a week? _______________

13. Would you say pain is or has been one of your major complaints that has brought you to this centre?

1. Yes
2. No

14. Did you/do you receive medicines:

a) To control severe pain?

1) Yes – where did the medicines come from or where did you receive it?

2) No (Go to No 15)

b) For other symptoms?

1) Yes – where did the medicines come from or where did you receive it?

2) No (Go to No 15)

15. Why don’t you get the medicines that you need for your pain? ***(The respondent can give more than one response. Mention the reason and ask whether that is the one or it is not the one)***

1) Medicines are very expensive

2) Medicines not available even when one has the money to buy them

3) I do not know the medicines to buy

4) Any other; Specify _________________

16. Apart from medicines, what other services are you given in this organisation? ***(The respondent can give more than one response)***

1) Counselling/psycho-social support

2) Legal advice e.g. to make a will

3) Spiritual talks from religious leaders

4) Others, (Specify)_______________________________________________

**Section C: Ethical issues**

17. Are you given information about side effects of the medicines you are given?

1) Yes (Go to No 19)

2) No

18. Why do you think you have not been given that information? ***(The respondent can give more than one response)***

_____________________________________________________________________________________

**Section D: Legal issues**

19. Do you know what a will is?

1) Yes 2) No

20. Have you received any advice on succession planning?

1) Yes 2) No

Thank you for your time!
