# Supplementary material for: The legal needs of people receiving palliative care in Uganda: A multi-method assessment to advance universal health coverage
Source: Palliat Care Soc Pract. 2025 Jun 26;19:26323524251347652. doi: 10.1177/26323524251347652 (PMC12202919; doi:10.1177/26323524251347652)
Supplement: sj-docx-2-pcr-10.1177_26323524251347652 – Supplemental material for The legal needs of people receiving palliative care in Uganda: A multi-method assessment to advance universal health coverage [file sj-docx-2-pcr-10.1177_26323524251347652.docx]

**S2 Appendix:**

**Key Informant Interview Guide for Healthcare Providers**

**ID :**

Gender (male/female) :

Job Title :

Organisation/Health facility :

Date of Interview :

Name of interviewer :

Time started-Ended :

Transcribed by :

*General introduction – ask questions such as length of time in job, specific role, etc.*

1. ***Introductory questions***
   - - 1. What services do you offer at this facility? *(probe for legal support, spiritual support, palliative care)*
       2. At what stage do most patients come to this facility? (*Probe whether they come early enough or if they come when they are very sick. Is there a difference between when men and women come?)*
       3. What is the catchment area for your patients/where do you patients come from?

*Probe into differences for men and women in how far they travel.*

1. ***Rights***
   - - 1. Are you aware of human rights related to the patients you serve? What are they? Would you say that patients are aware of their rights? How do you address these rights?
2. ***Laws and conventions***
   - - 1. Are you aware of international laws and conventions in relation to the patients you serve? Do you find them applicable or even necessary in your setting? Explain your response. (Probe with examples)
       2. In your experience, are there legal issues that affect the patients you serve? In which way do they affect the patients? Give detailed explanation.

*Probe into gender differences for legal issues.*

1. ***Death, dying and the law***
   - - 1. Have any of your patients ever sought guidance from you or your staff on:

a) How to make a will

b) Guidance on how to prepare for their end of life

c) Worries about life after their death.

*Probe into whether men or women seek guidance more.*

1. How do you involve families in the care of the patients, and *do* you think the families are usually more or less prepared to handle bereavement *and after life* issues such as ownership of property, care of orphans, etc.?

*Have you been involved in the discussion of any of the above issues with any of your patients?* Give detailed explanation.

1. ***Ethical issues***
2. As a practitioner, what are some of the ethical considerations you consider take into account providing care for patients?
3. ***Access issues***
4. What palliative care medicines do you have at this facility? How accessible (in terms of availability and affordability) are the medicines you use in providing palliative care?

*Probe for availability of opioids.*

1. In general, how do health providers (doctors, clinical officers, nurses, midwives) in this facility and generally in the country get training or education in pain control medication and methods?
2. What control measures are in place at this facility to ensure that palliative care medicines such as opioids do not get into hands of unlawful/illicit/illegal users?

*Probe into legal requirements for opioids.*

1. Which staff cadres prescribe opioids at this facility? Are opioids prescribed effectively for all patients or are there any recommendations you would make for change? *Explain.*
2. What do you consider the biggest barrier to prescribing and/or dispensing opioid medications to persons under palliative care?

*Probe whether it is the lack of training of health workers, the legal system, the drug policy in the country, the cost of the medicines, and the fear of using the medicines by the patients. Are the barriers for men different from those of women?*

1. Do you have any questions on what we have discussed?

Thanks very much for your time!
