# Supplementary material for: The legal needs of people receiving palliative care in Uganda: A multi-method assessment to advance universal health coverage
Source: Palliat Care Soc Pract. 2025 Jun 26;19:26323524251347652. doi: 10.1177/26323524251347652 (PMC12202919; doi:10.1177/26323524251347652)
Supplement: sj-docx-3-pcr-10.1177_26323524251347652 – Supplemental material for The legal needs of people receiving palliative care in Uganda: A multi-method assessment to advance universal health coverage [file sj-docx-3-pcr-10.1177_26323524251347652.docx]

**S3 Appendix:**

**Key Informant Interview Guide for Legal Expert Professionals Working in Uganda’s Medicines Supply Chain**

**ID**  **:**

Gender (male/female) :

Occupation :

Any additional professional training :

Organisation :

Position in the organisation :

Date of Interview :

Name of interviewer :

Time started-Ended :

Transcribed by :

*Note the interviewer will introduce to the officer what palliative care is and the role opioids in life-threatening illnesses.*

1. ***Medical regulations***
2. Tell me about how laws, policies, and regulations concerning clinical use of opioid medicines are enforced?

*Prompts: Elicit details of the day-to-day practice of enforcement.*

1. Do laws, regulations and policies relating to clinicians create any additional restrictions for prescribing opioids? Tell me about them.

*Prompts: Elicit each barrier, for example, do regulations…*

- 1. Limit the length of prescription validity or refill?
  2. Require record keeping of prescriptions?
  3. Require that a patient be “terminal” for pain relief?
  4. Require a particular diagnosis?
  5. Limits on dosage or type of drug?
  6. Limits dispensing authority?

*Probe for differences by drug type or dose.*

1. ***Dispensing regulations***
2. Tell me about how laws, polices and regulations concerning health providers who dispense opioid medicines are enforced?

*Prompts: Elicit details of the day-to-day practice of enforcement.*

1. Are health workers subject to inspection, review, or additional scrutiny for, or related to, prescriptions of opiates or opiate substitutes?
2. Do licensing and disciplinary bodies review patterns and “legitimacy” of pharmacist’s dispensing of opioids?
3. ***Palliative care in general***
4. What do you consider to be the legal needs of the family and those left behind which should be taken care of before the person dies?
5. What are the legal issues we need to consider when caring for people with life-threatening illnesses?

Thank you for the time!
