# Supplementary material for: The legal needs of people receiving palliative care in Uganda: A multi-method assessment to advance universal health coverage
Source: Palliat Care Soc Pract. 2025 Jun 26;19:26323524251347652. doi: 10.1177/26323524251347652 (PMC12202919; doi:10.1177/26323524251347652)
Supplement: sj-docx-4-pcr-10.1177_26323524251347652 – Supplemental material for The legal needs of people receiving palliative care in Uganda: A multi-method assessment to advance universal health coverage [file sj-docx-4-pcr-10.1177_26323524251347652.docx]

**S4 Appendix:**

**Key Informant Interview Guide for Legal and Human Rights Practitioners and Experts**

**ID :**

Gender (male/female) :

Occupation :

Any additional professional training :

Organisation :

Position in the organisation :

Date of Interview :

Name of interviewer :

Time started-Ended :

Transcribed by :

*Note the interviewer will introduce to the officer what palliative care is and the role of opioids in life-threatening illnesses.*

1. **Adequacy of the human right to palliative care in the law**
2. For how long have you been in practice as a human rights defender/advocate?

*Probe time and what he/she has mainly been doing for the past 5 years.*

1. In your opinion and experience, do you feel there is an adequate guarantee of the human right to health and, specifically, to palliative care in the law?
   1. Which laws are you referring to?
   2. If yes (No. 2), briefly state the reasons for your answer.

1. What would you describe as the legal needs of persons receiving palliative care? What legal support do they have currently?
2. In your view, does the law sufficiently address the legal needs of the various interest groups, including children, the elderly, disabled people, prisoners, refugees with life-threatening illnesses, etc.? Can you specify the needs under each group? *Ask to explain in detail.*
3. Do you feel the current laws adequately protect the rights of persons with life-threatening illnesses?
   1. If not, give reasons?
   2. What changes would you recommend?
4. **Succession, guardianship, and other legal issues associated with the end of life**
5. Do you help people in writing/drafting of wills?
   1. Has any person with a life-threatening illness ever instructed you to write a will?
   2. What was his or her basic motivation/reason for a will?
   3. In your opinion, do you think people with life-threatening illnesses should make wills?
   4. Why? *Give reasons for your answer*.
6. Would you or your organisation provide *pro bono* services to people with life-threatening illnesses?
7. In your experience as a legal practitioner, are there any problems a family may face when an influential family member dies without making a will?
   1. If yes, state the problems such a family may face?
8. **Estate/property management during illness**
9. In your view, what other available avenues do you feel persons receiving palliative care can use to manage their properties or for debt management, family, or planning for the life ahead of them? *Probe for powers of attorney if not mentioned.*
10. **Social security/benefits etc. as an aspect of palliative care**
11. Are people with life-threatening illnesses entitled to any form social benefits from the Government under the Constitution or any other law that you know?
    1. If not, what do you propose should be done?
    2. What would you propose should be done to address the various interest groups; namely, the children, elderly, prisoners, etc., receiving palliative care?
12. What other legal reforms do you propose to ensure realisation of a fully-fledged palliative care in Uganda?
13. **Ethics and palliative care; examine the relationship**
14. In your view, do feel there are adequate laws to regulate the practice of medicine, pharmacy, and nursing in Uganda?
    1. How does that impact on the rights of people with life-threatening illnesses?
15. Do you have any questions for us?

Thank you for your time!
